# Hydrogels for Biomedical Applications: Cellulose, Chitosan, and Protein/Peptide Derivatives

**DOI:** 10.3390/gels3030027

**Published:** 2017-07-17

**Authors:** Luís J. del Valle, Angélica Díaz, Jordi Puiggalí

**Affiliations:** Barcelona Research Center for Multiscale Science and Engineering, Universitat Politècnica de Catalunya, Escola d’Enginyeria de Barcelona Est-EEBE, c/Eduard Maristany 10-14, Barcelona 08019, Spain; luis.javier.del.valle@upc.edu (L.J.d.V.); angelicadiaz07@gmail.com (A.D.)

**Keywords:** cellulose, chitosan, collagen, gelatin, peptides, self-assembling, nanogels, shape memory, molecularly imprinting, 3D printing

## Abstract

Hydrogels based on polysaccharide and protein natural polymers are of great interest in biomedical applications and more specifically for tissue regeneration and drug delivery. Cellulose, chitosan (a chitin derivative), and collagen are probably the most important components since they are the most abundant natural polymers on earth (cellulose and chitin) and in the human body (collagen). Peptides also merit attention because their self-assembling properties mimic the proteins that are present in the extracellular matrix. The present review is mainly focused on explaining the recent advances on hydrogels derived from the indicated polymers or their combinations. Attention has also been paid to the development of hydrogels for innovative biomedical uses. Therefore, smart materials displaying stimuli responsiveness and having shape memory properties are considered. The use of micro- and nanogels for drug delivery applications is also discussed, as well as the high potential of protein-based hydrogels in the production of bioactive matrices with recognition ability (molecular imprinting). Finally, mention is also given to the development of 3D bioprinting technologies.

## 1. Introduction

Hydrogels are three-dimensional polymer matrices able to retain large amounts of water in a swollen state, a feature that makes them similar to biological tissues. In fact, biomedical applications of hydrogels have been explored continuously since the 1960s, when they were first discovered [1]. Physical and chemical crosslinks are fundamental to building a hydrophilic network in which chemical agents can also be incorporated, giving rise to drug delivery systems and even to new functional materials. In fact, research on hydrogels is nowadays mainly focused on developing stimuli-responsive smart materials and hydrogels with shape memory properties that could be applied for innovative biomedical uses [2,3,4,5]. Different reviews have addressed natural and synthetic formulations as well as the corresponding general applications [6,7,8,9]. 

Great effort is directed towards designing molecules capable of promoting molecular aggregation (gelators) [10,11]. The incorporation of units able to establish one-dimensional hydrogen bonding interactions such as amides and saccharides merits attention. In fact, derived low molecular weight compounds can be heated in an appropriate solvent to form a supersaturated solution, which after cooling to room temperature can give rise to a gel through an aggregation process [12,13,14]. Small molecules can be organized into polymer-like fibers that become entangled and constitute a continuous matrix that entraps the solvent by surface tension. A heterogeneous “solid” matrix is obtained with a hierarchical superstructure aggregation that creates dimensions from the nanometric to the micrometric scale.

Development of in situ gelling polymeric matrices is also of great interest in tissue regeneration since these materials can be used as injectable hydrogels. These can act as cell vehicles that have the ability to take the shape of the corresponding tissue cavity. Furthermore, problems related to cell adhesion can be minimized since cells can directly be incorporated into the injectable solution [15,16]. A suitable in situ hydrogel for biomedical applications should be soluble in aqueous media and have a fast sol-gel transition under physiological conditions without releasing toxic byproducts or harming surrounding tissue [17]. In general, injectable hydrogels are designed with functional groups sensitive to external stimuli such as pH, temperature, and light [18].

The present review is basically focused on the study of hydrogels for biomedical applications, mainly restricted to cellulose and chitosan (a chitin derivative) as the most abundant natural polymers on earth and collagen as the most abundant protein in the human body. Attention is also paid to the use of peptides due to their abovementioned self-assembling properties. Different subsections also introduce the most relevant topics that can nowadays be considered: (a) responsiveness of hydrogels to external stimuli; (b) development of micro and nanogels (i.e., micro or nanoscopic three-dimensional networks comprising cross-linked polymer molecules dispersed in a proper solvent); (c) shape memory hydrogels; (d) molecularly imprinted hydrogels and (e) protein-based hydrogels for 3D printing.

## 2. Hydrogels Derived from Cellulose

**Figure 1:**
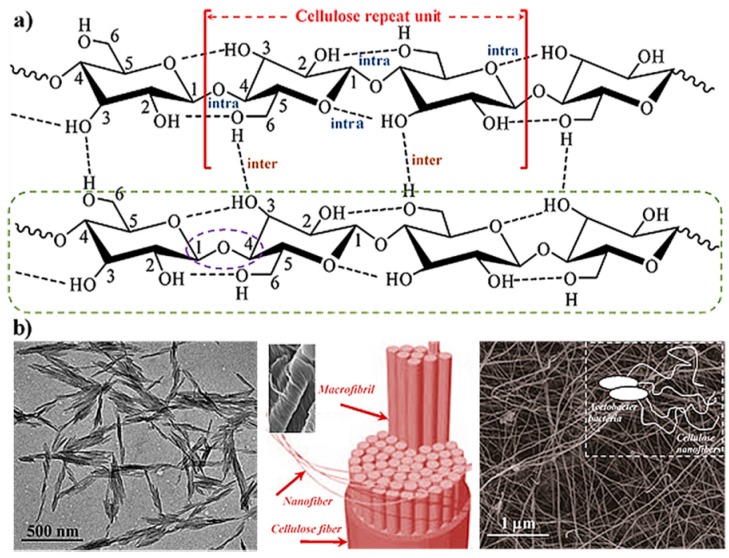
(**a**) Scheme of the linear molecular chain (green box), the syndiotactic repeat unit (garnet), the establishment of glycosylic bonds between glucose rings (violet ellipsoid) and intra and intermolecular hydrogen bonding interactions; (**b**) TEM micrograph of cellulose nanowhiskers (left), scheme and SEM micrograph of nanofibers derived from a fiber of cellulose (middle) and TEM micrograph of bacterial cellulose (right). Reproduced with permission from [19], copyright 2007 ACS; and reproduced from [20].

Cellulose is the most abundant biopolymer, mainly as a consequence of its properties that make it an essential structural component of green plants, marine animals, algae, and bacteria. Sustainability, biodegradability, biocompatibility, and low cytotoxicity are other characteristics that justify the development of a great number of applications in the biomedical field that concern cellulosic materials. From a chemical point of view, cellulose is defined by the connection through β-(1→4) glycosidic bonds between D-glucose units (Figure 1), which gives rise to a linear syndiotactic polymer with hydroxyl groups arranged in an equatorial disposition. The molecular chain can be visualized as a stiff rod-like conformation that can be arranged, giving rise to crystalline fibrous materials. Strong intra- and intermolecular hydrogen bonding interactions can be found, with different crystalline structures of cellulose reported depending on its origin (e.g., cellulose I_α_ and cellulose I_β_ for polymers produced by bacteria and plants, respectively) and chemical treatments (e.g., cellulose II for regenerated fibers).

Cellulose can be employed to produce hydrogels for biomedical applications according to two differentiated methodologies: (a) the use of cellulose-based matrices and (b) the use of composites incorporating nanocellulose.

Cellulose based-hydrogel matrices are nowadays ideal materials for tissue engineering applications [21] due to intrinsic properties like non-toxicity, biocompatibility, tunable and porous microstructure, and good mechanical properties [22]. Applications of cellulose derivatives can be enhanced when blends or hybrids with other components like chitosan are considered [23]. 

Nevertheless, cellulose-based hydrogels have as a main limitation the low solubility of cellulose in both water and most organic solvents due to the hydrogen-bonded structure [24]. This problem can be avoided via chemical modification and specifically by the conversion of the hydroxyl pendant groups into ether and cationic groups, although it should be taken into account that final properties can be negatively altered. Research has also been focused on directly dissolving cellulose in appropriate non-toxic solvents. In this sense, alkali/urea aqueous systems appear highly promising [25,26] since inclusion complexes can be formed at low temperatures, which demonstrates the possibility of preparing membranes and hydrogels from these media [27,28].

Hydrogels derived from cellulose can be prepared by the crosslinking of aqueous solutions of cellulose ethers (e.g., methylcellulose (MC), ethylcellulose (EC), sodium carboxymethylcellulose (NaCMC), or hydroxypropyl methylcellulose (HPMC)). MC is ideal for the preparation of thermoresponsive hydrogels due to its hydrophobic–hydrophilic equilibrium, which gives rise to a collapse or an expansion of molecular chains by small temperature changes around its critical value [29].

Nanocellulose is a general term that defines a nanostructured material that comprises cellulose nanocrystals (CNC), cellulose nanofibers (NFC), and bacterial cellulose (BC). Nanocrystals with a whisker morphology (Figure 1b) can be easily produced by the treatment of cellulose with strong acids [30] that cause the degradation of amorphous regions and produce whisker-like crystals to be used as fillers in bio-based matrices [31]. Cellulose nanofibers (NFC) (Figure 1b) are produced by mechanical treatments of natural fibers (e.g., high pressure and ultrasonic homogenization, grinding, and microfluidization). Clinical applications of NFCs have been justified considering their cytocompatibility and also the tolerogenic potential in the immune system [32]. Finally, bacterial or microbial cellulose is directly produced by bacteria (e.g., *Acetobacter* strain) from glucose residues. The polymerized material is secreted from the cell and crystallizes, giving rise to nanofibers with diameters smaller than 100 nm (Figure 1b) [33]. These nanofibers can be connected, forming a 3D networked structure. 

CNCs can display interesting effects on the gelation mechanism of hydrogels and can improve mechanical properties and dimensional stability and even favor the drug release [34,35,36]. CNCs have also been employed as nanofillers to improve, for example, the compression modulus (up to 92 kPa for a load of 20 wt %) of hybrid hydrogels based on gelatin and alginate [37]. Hydrogels composed of an interpenetrating network of sodium alginate and gelatin reinforced with 50 wt % of CNCs have also been developed for cartilage applications. Specifically, modulus, strength, and strain values of 0.5 GPa, 14.4 MPa, and 15.2%, respectively, were attained, with the modulus clearly higher than that determined for natural cartilage. The double cross-linked system was prepared by a freeze-drying process, with the carboxyl surface groups of CNC contributing to the enhancement of mechanical properties and structural stability [38]. 

Surface modification of CNCs can improve their performance since they cannot only be used as fillers but also as cross-linking agents that increase the adhesion of the filler with the polymeric matrix [39]. Thus, aldehyde-functionalized CNCs have been employed as cross-linkers for carboxymethylcellulose hydrogels [40]. Maleimide-functionalized CNCs have also been proposed as effective cross-linkers for the formation of hydrogels based on gelatin and chondroitin sulfate, which are stiffer networks with lower swelling ratios [41]. 

NFC hydrogels can be prepared from suspensions of NFC fibrils produced by a simple mechanical treatment, but nowadays better results have been described when fibrils with a high negative charge on their surface are employed. Thus, oxidation processes such as the treatment with 2,2,6,6-tetramethylpiperidine-1-oxyl radical (TEMPO) produced more stable dispersions/suspensions than hydrogels formed under limiting values of polymer concentration and charge density [42,43]. Great efforts have also been developed to control the porosity and network microstructure to ensure the transportation of nutrients and waste products when tissue engineering applications are considered [44]. Thus, hydrogels with controllable swelling degree, porosity, and surface area can be prepared by tuning the charge density of fibers, the conditions of the swelling media, and the processing methodology [45]. In general, NFC hydrogels have low mechanical properties (e.g., a storage modulus close to 10 Pa was reported for a native NFC hydrogel at 0.5 wt % [46]) and therefore applications are usually limited to soft tissues [47,48]. The use of reinforcing agents has been proposed in order to improve mechanical properties since they can easily be incorporated into NFC hydrogels due to their high degree of swelling. Furthermore, the addition of molecules such as hemicellulose can provide additional benefits like anticancer and antioxidative properties [49,50]. Different hemicelluloses (e.g., galactoglucomannan, xyloglucan, and xylan) have been studied as crosslinkers to tune the structural and mechanical properties of NFC hydrogels, as well as to study their effect on cell behavior (adhesion, growth, and proliferation) during wound healing processes [51]. Nanocellulose charge density was found to be a determining factor for the incorporation of hemicellulose, the derived surface (topography and roughness) and the mechanical and biological properties of the composite hydrogels. 

BCs are currently used as promising hydrogels for the development of functional nano-biocomposites [52,53,54]. Furthermore, derived materials can display appropriate mechanical properties when being used as membranes. Composite systems with collagen were, for example, evaluated in order to promote cell adhesion and viability. Specifically, BC membrane surfaces were coated with collagen and alginate on each side to favor cell adhesion and protect transplanted cells from immune rejection, respectively [55]. It was also demonstrated that cells were able to release dopamine through the BC composite membrane, a promising feature for its use as a material for cell encapsulation.

The high purity and hydrophilicity of bacterial cellulose make it a promising material for wound-healing applications [56], with different products already commercialized for dressings (e.g., XCell, Biofill, or Dermafill) [57,58].

## 3. Hydrogels Derived from Chitosan 

Chitin (poly-(1→4)-*N*-acetyl-glucosamine) is one of the most abundant natural polymers since its facility to produce microfibrils makes it an essential structural component of cell walls (e.g., fungi and yeast) and of the exoskeleton of many invertebrates (e.g., shrimps and crabs). This polysaccharide is characterized, like cellulose, by a β-(1→4) glycosidic bond and can be transformed into the water-soluble chitosan (CS) upon deacetylation in strong alkaline solutions. The high availability of chitosan, its biodegradability and biocompatibility have enhanced interest in its use as a hydrogel with improved structural stability and high capability to absorb water. The physical properties of chitosan can be controlled by changing the molecular weight of the precursor, the degree of depolymerization, and deacetylation, and finally by modifying the interactions that can be established with both hydroxyl and amine groups that are present in the molecular backbone. The cationic character of CS favors the formation of gel particles through electrostatic interactions [59,60], with, e.g., sodium sulfate employed as a precipitant [61]. CS molecules can also interact with hydrophobic components, giving rise to amphiphilic particles with great self-assembly and encapsulation ability. Interactions established between chitosan and drugs should be appropriate to produce the expected pharmacological effect at the target site. 

Chitin has also been extensively considered for tissue engineering and drug delivery applications; specifically, it has been processed in the form of hydrogels and nanogels despite the disadvantages associated with its water insolubility [62,63]. Nevertheless, it should be taken into account that biological properties (e.g., antimicrobial, hemostatic, or mucoadhesion, effects) decrease with the degree of acetylation. Several attempts have been proposed to get three-dimensional sponge-like materials from chitin [64,65]. These scaffolds favor the deposition of the extracellular matrix on chondrocytes and have applications in cartilage tissue engineering [66]. Preparation of chitin hydrogels is feasible by employing a mild medium based on a supersaturated CaCl_2_–methanol solution [67]. Hydrophilic chains are able to aggregate through intermolecular interactions and form a network-like structure [68]. Chitin nanogels have been employed to release anti-cancer drugs (e.g., doxorubicin [69]), anti-fungal drugs (e.g., fluconazole [70]), and proteins (e.g., bovine serum albumine (BSA) [71]) [72].

The mucoadhesion property of chitosan gels has enhanced interest in using them as drug delivery systems since the bioavailability of loaded bioactive drugs can be increased [59]. Furthermore, CS hydrogels can be processed in the form of micro-/nano-sized spherical particles or beads where bioactive compounds can be encapsulated. These beads swell in acidic media, facilitating drug release. An additional advantage of chitosan beads is their relative high hydrophobicity, which facilitates intestinal absorption [73]. Delivery systems based on chitosan have been developed for the treatment of colon [74,75] and hepatic [76,77] diseases. Several hydrogels based on chitosan have also been designed to encapsulate radioisotope drugs for site-specific cancer therapy. These chitosan hydrogels can display photo-responsiveness and a thermoreversible gelling capacity [78,79].

Hydrogels composed of chitosan have the capacity to adsorb both anionic and cationic molecules if hydrogen bond interactions can still be established. This feature confers on CS-based hydrogels a great potential in fields as diverse as water purification and protein encapsulation [80].

**Figure 2:**
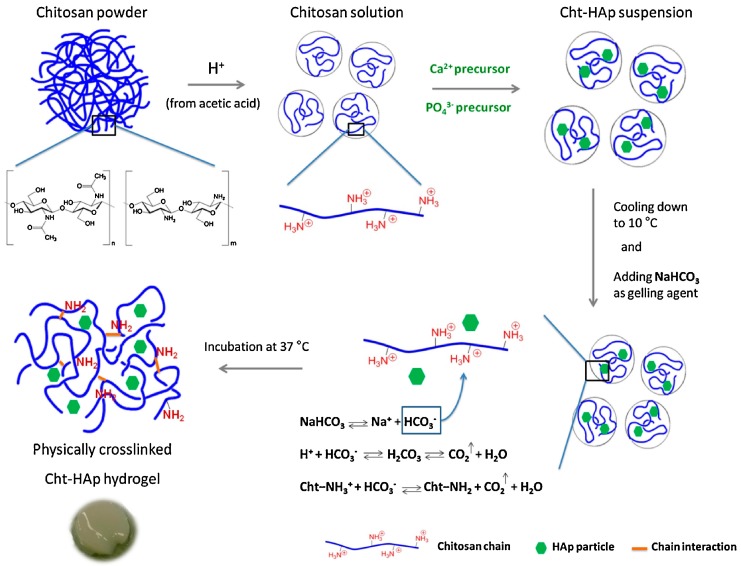
Preparation of a physically crosslinkinked injectable hydrogel based on chitosan and hydroxyapatite. Reproduced with permission from [87], copyright 2017 Elsevier.

Most injectable hydrogels for biomedical applications are based on CS, it being possible to modify the composition in order to undertake chemical or physical gelling by UV irradiation or the increase of temperature or pH [81,82]. Probably, the main systems studied are those based on the addition of a glycerophosphate salt that initiated the sol-gel transition at body temperature [83] and also the additional incorporation of genipin as a crosslinking agent [84]. Incorporation of hydroxyapatite has also been revealed to be useful for bone tissue regeneration since it enhanced cell adhesion and proliferation and improved osteogenic properties [85,86]. A pH-responsive CS-based injectable hydrogel incorporating hydroxyapatite has been prepared using sodium bicarbonate (NaHCO_3_) as the gelling agent (Figure 2). This system provided a neutral environment suitable for cell encapsulation and allowed non-cytotoxic, fast gelation (e.g., 4 min). Physical crosslinks were the consequence of glucosamine deprotonation and produced materials with good resistance to applied shear deformation [87].

**Figure 3:**
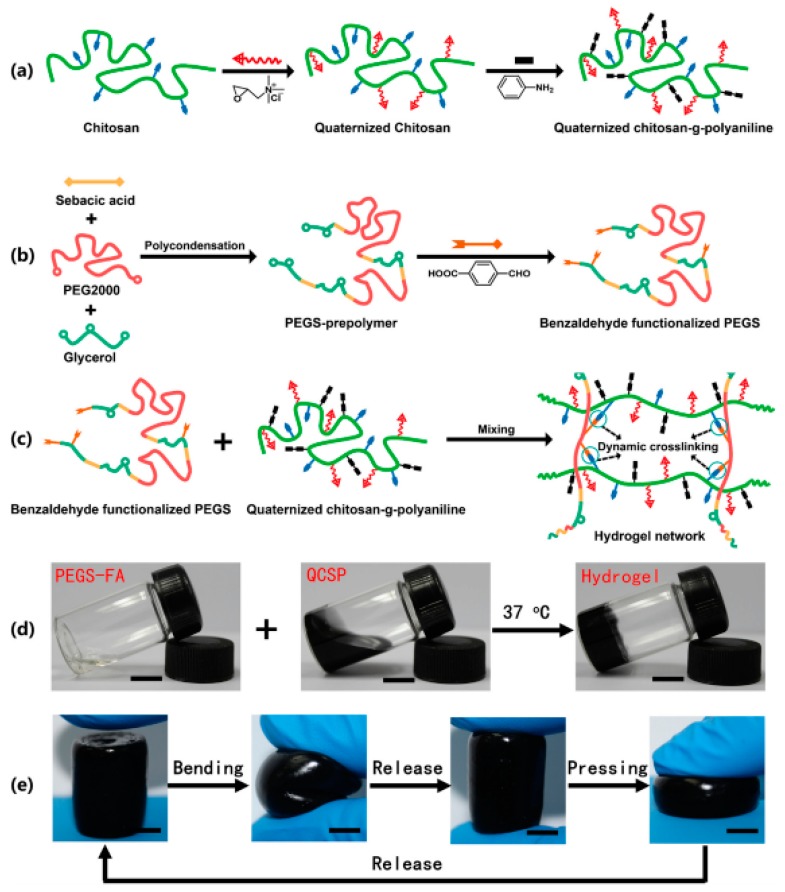
Scheme showing the synthesis of chitosan-*g*-aniline (**a**), PEGS-FA copolymers (**b**) and the structure of the hydrogel derived from both copolymers (**c**). Photographs showing the corresponding solutions (**d**) and flexible behavior of the hydrogel under bending and pressing efforts (**e**). Reproduced with permission from [89], copyright 2017 Elsevier.

Quaternized chitosan-*g*-polyaniline copolymers have been synthesized [88] in order to enhance the antibacterial activity and cytocompatibility of CS. The grafted copolymer was postulated as an idoneous injectable hydrogel dressing [89] due to its good biocompatibility, bactericidal effect, conductivity, and good free radical scavenging capacity [90]. Specifically, injectable hydrogel dressings were prepared at physiological conditions by mixing solutions of quaternized chitosan-*g*-polyaniline with a poly(ethylene glycol)-*co*-poly(glycerol sebacate) copolymer having benzaldehyde functional groups (PEGS-FA) (Figure 3). Soft and flexible hydrogels were derived as a consequence of the chain flexibility given by PEGS-FA and the dynamic network of chemical bonds. The final conductivity was a result of ionic conductivity from amino groups and ammonium groups, and electronic conductivity from doped polyaniline. Conductivity varied between 3.13 mS/cm to 2.25 mS/cm as the crosslinker concentration increased from 0.5 to 2 wt %. A crosslinker concentration of 1.5 wt % was found to be optimal for enhancing blood clotting capacity and the in vivo wound healing process. 

pH-Sensitive CS hydrogels reinforced with CNCs were prepared using glutaraldehyde as a crosslinker due to its high reactivity with chitosan amine groups [91]. CNCs were incorporated in the preformed polymer network, giving rise to hydrogels characterized by a combination of amorphous and crystalline phases and the increase of compression modulus from 25.9 ± 1 to 50.8 ± 3 kPa when the nanocellulose content was 2.5 wt %. The maximum swelling ratio was found for a pH of 4.01, where chitosan amine groups were protonated and hydrogen bonds became consequently dissociated. The CS/CNC hydrogel was also interesting for drug release and specifically for the delivery of curcumin [92]. 

Blending cellulose with chitosan may give rise to hydrogels with improved properties, but it is problematic to get a homogeneous aqueous solution of both components since they need alkaline (cellulose) and acidic (chitosan) aqueous media. Nevertheless, a water-soluble chitosan derivative (i.e., hydroxyethyl chitosan) has successfully been employed to make porous scaffolds using silicon dioxide particles as a porogen and following a freeze-drying process [93]. Good overall performance and an ability to reach the equilibrium swelling state were observed. 

## 4. Hydrogels Derived from Collagen and Gelatin

Collagen is the most important protein that forms part of the extracellular matrices. It can be obtained from skin and other tissues by enzymatic and acidic treatments. A hydrogel can be produced after neutralization of the acid solution and subsequent heating to body temperature. Gelatin is derived when the typical triple helix of collagen is broken into a single molecule that could undergo a reversible sol-gel transition at room temperature [94]. Gelatin exhibits biocompatibility but its applications are hindered by its low mechanical properties, which usually make the establishment of additional crosslinks necessary. Non-toxic enzymes (e.g., transglutaminase and tyrosinase) are usually preferred [95,96] as new cross-linking agents. Suitable hydrogels, mainly used as vehicles for cell transplantation (e.g., mesenchymal stem and stromal cells), can therefore be prepared [96,97].

Multiple studies have demonstrated that collagen can play a highly positive role in tissue regeneration [98,99,100], but its use in biomedical applications is somehow limited by its poor mechanical properties and high degradation rate [101]. Therefore, blending collagen with other polymers has been postulated as an alternative solution to improve final performance [102]. In this way, protonated CS appears to be an ideal polymer to interact with negatively charged collagen. Thermoresponsive hydrogels based on CS and different types of collagen have been studied. They have potential for biomedical uses as matrices for the encapsulation of cells, repair of bone defects, and promotion of in vivo cell differentiation [103,104,105]. Hydrogels containing CS, acid-soluble collagen (ASC), and glycerophosphates have recently been prepared [106] for tissue regeneration, having demonstrated good biocompatibility and the ability to support the survival and proliferation of encapsulated cells. 

**Figure 4:**
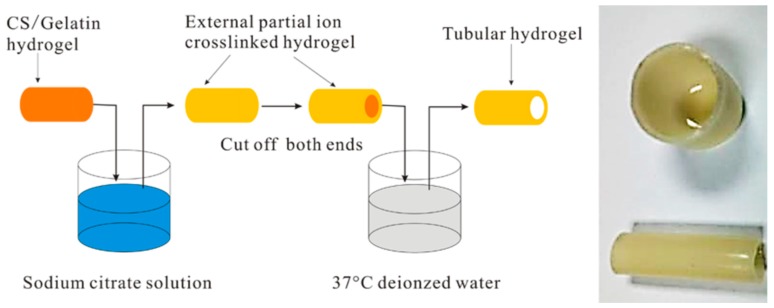
Preparation of hollow structures (e.g., cup and tube) from CS/gelatin hydrogels based on a controllable ion crosslinking process. Reproduced with permission from [113], copyright 2017 Elsevier.

High-strength hydrogels based on BC and gelatin and with good biocompatibility have been studied [107]. The preparation process was difficult since it involved three steps, and it was consequently necessary to develop similar systems with easier processing than the two-step method applied for dual-crosslinked chitin and cellulose hydrogels [108,109]. Development of multiple crosslinked structures therefore appears to be a suitable option for producing hydrogels with good mechanical performance and biocompatibility. Efforts are also focused on new processes that ensure shape designability and good formability. Namely, we try to avoid the use of injectable hydrogels that require the modification of raw materials, which has usually led to materials with low mechanical properties. On the other hand, tubular hydrogels hold great interest for delivery applications, exchange channels for oxygen and nutrients, and vascular repair [110,111,112]. Wu and collaborators proposed a mild interrupted ion crosslinked process, which allowed for obtaining hollow hydrogels with a controllable shape [113]. Non-stable CS/gelatin hydrogels were first obtained by the aggregation of the gelatin helix domains, but a subsequent treatment with a sodium citrate solution produced a stable, physically crosslinked network. The efficiency of this step was dependent on the immersing time in the solution (i.e., the capability of ions to diffuse into the hydrogel). Therefore, at low times it was possible to melt/dissolve the non-ionic crosslinked core by exposure to deionized water at 37 °C (Figure 4). The process can be combined with thermal welding and etching methods to program the external shape of complex hydrogel architectures. 

Development of conductive hydrogels is highly interesting for cardiac regeneration and repair. Natural hydrogels derived from collagen and gelatin [114,115] have usually been considered to support cardiac cell functions despite having an insulating character. This shortcoming could be avoided by using electrically conductive nanomaterials. Gold nanostructures have several advantages like high conductivity, easy modification and fabrication, minimum cytotoxicity, and varied architecture (e.g., nanowires, nanorods, and nanoparticles). In any case, hydrogel matrices should be designed to encourage good cell adhesion, a feature that can be attained with gelatin-based hydrogels. Specifically, hybrid hydrogels composed of a UV-crosslinkable gelatin methacrylate and incorporating gold nanorods have recently been found to be appropriate for cardiac tissue engineering [116].

Carbon nanotubes (CNTs) [117] are also considered for enhancing conductivity, although several limitations concerning cytotoxicity and a complex fabrication procedure have been indicated [118,119]. In any case, considerable research has been carried out to develop conductive hydrogels based on CNTs. For example, the bulk electrical properties of collagen can be increased by the addition of single-walled carbon nanotubes (SWCNT). These clearly influenced neurite extension and enhanced neurite outgrowth, leading to suitable hydrogels for nerve regeneration [120].

## 5. Peptide Hydrogels

**Figure 5:**
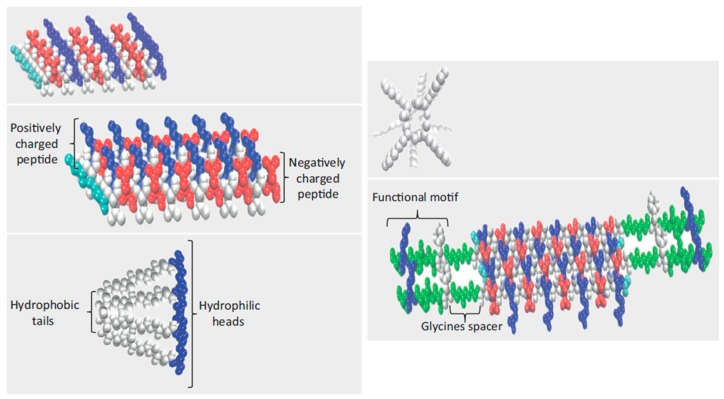
Typical structures of different self-assembled peptides: RADA-like SAPs, complementary coassembling peptides, peptide amphiphiles, cyclo-SAPs, and functionalized SAPs. Reproduced with permission from [121], copyright 1995 Elsevier.

Natural fibrillar proteins of the extracellular matrix (ECM) can be mimicked by the self-assembling of fully synthetic peptides (SAPs). A porous network having cell-binding sites or functional motifs can be formed and used to induce the growth and differentiation of host cells or alternatively as carriers for transplanted cells. Peptidic sequences can be assembled, giving rise to a variety of morphologies (e.g., nanofibers, nanotubes, nanovesicles, or nanoparticles), it being possible in some cases to trigger the assembly by modifying pH or temperature or by the presence of external cations. Different types of self-assembly can be considered (Figure 5): (a) alternate disposition of charged hydrophilic and hydrophobic residues (e.g., peptides based on the Arg-Ala-Asp-Ala sequence named RADA-like SAPs); (b) complementary co-assembling peptides (CAPs); (c) peptide amphiphiles; (d) cyclic peptides; and (e) functionalized peptides.

Organized stable β-sheet superstructures are characteristic of RADA and similar sequences. Specifically, Ac-(RARADADA)_2_-CONH_2_ [122] and Ac-(RADA)_4_-CONH_2_ [123] are composed of 16 amino acids. They form structures in aqueous media with charged amino acid side groups oriented on one side of the sheet and the hydrophobic side groups on the other side (i.e., a hydrophobic inner pocket was derived). The resultant scaffolds are appropriate for 3D cell culture, wound healing, and synapse growth. In fact, the indicated sequence has great similarity with the well-known RGD cell adhesion motif. 

CAPs are based on the attraction and self-repulsion of two peptides having opposite electric charges [124]. For example, mixing of positive Ac-(LKLH)_3_-CONH_2_ and negative (Ac-(LDLD)_3_CONH_2_ spontaneously formed double layers of β-sheets and gave rise to 3D molecular assemblies composed of nanofibrillar structures, as is also typical for the above indicated SAPs. Derived hydrogels from CAPs can retain 95–99% of water and had pores between 5 and 200 nm [125]. CAPs avoid the use of pH shift as a triggering stimulus, which may be problematic in some biological conditions.

Peptide amphiphiles are similar to the phospholipids existing in membranes since they are composed of hydrophobic tails and hydrophilic heads. The former are based on nonpolar amino acids with different degrees of hydrophobicity, while the latter can be based on positively or alternatively negatively charged amino acids. 

Self-assembled tubular structures are usually derived from the stacking of cyclic peptides. These structures are stabilized by hydrogen bonds that are established between carbonyl and amide groups. These interactions are directed perpendicularly to the ring, while the amino acid side groups are directed outward [126]. 

**Figure 6:**
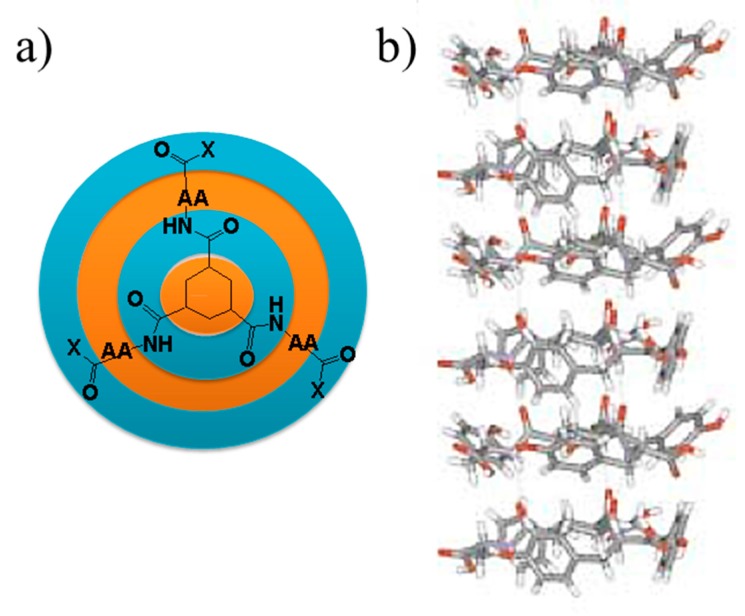
(**a**) Scheme showing the hydrophobic (blue) and hydrophilic (orange) regions of a cyclohexane-based hydrogelator having amino acids (AA) with hydrophobic side groups; (**b**) a single strand formed through the multiple hydrogen bonds that each single molecule can establish. Reproduced with permission from [127], copyright 2004 Wiley.

Amino acids and peptides may contain hydrophobic groups, which may provide hydrophobic interactions and create a synergistic effect with the more hydrophilic hydrogen bonding interactions. This point is illustrated in Figure 6, where a hydrogelator with good self-assembling ability was prepared by coupling three amino acids with hydrophobic side chains to a hydrophobic cyclohexane molecule. One-dimensional hydrogen-bonded stacks were derived, also taking advantage of the hydrophobic interactions established between the central cores and the shielding effect of the side chains over potential interactions between the amides and water molecules of the solvent [127].

In general, an ideal functionalized self-assembling structure should have: long alkyl chains that constitute the hydrophobic domain; a peptide sequence promoting β-sheet structures; charged amino acids to favor water solubility; and a bioactive epitope [128]. The hydrophobic interactions favor the development of self-assembled structures. Nevertheless, the final morphology can be influenced by the size of the hydrophobic moiety, there being, for example, a report of a morphological change from simple sheet stacking to long nanotubes and finally to short nanorods as the length increased [129]. 

## 6. Responsiveness to External Stimuli of Peptide-Derived Hydrogels for 3D Cell Culture

Control of supramolecular interactions that lead to the formation of assembled supramolecular structures may facilitate the in situ encapsulation of cells and even the subsequent delivery of the proliferated cells by switching the hydrogel to the sol state by means of proper external stimuli. 

The self-assembly of ionic peptides can be controlled through the pH of the solution. Thus, assembly is possible when the solution pH leads to a practically zero net charge of the peptide molecules. Ionic force also has a great influence in this assembling process since the counter-ions can decrease the charge distribution. Multiple examples can be mentioned concerning the encapsulation of cells (e.g., chondrocytes [130,131], human mesenchymal cells [132], or murine embryonic pluripotent stem cells [133]).

The switching between the hydrogel and solution phases can be controlled by the protonation and deprotonation of their amino and carboxylic terminal groups [134]. Short dipeptides with their amino terminal group blocked with the aromatic 9-fluorenylmethoxy-carbonyl group (Fmoc) were able to be dissolved in alkali solutions and form 3D hydrogels at neutral pH through hydrogen bonds and aromatic stacking interactions. In this way, cell dispersion could be mixed with a peptide solution before lowering the pH to physiological conditions. Cells like dermal fibroblasts were successfully encapsulated following the indicated procedure [135]. Peptides derived from serine and mainly from phenylalanine were also reported to produce such pH switchable hydrogels [136,137,138,139]. 

A method based on the change of polarity of the solvent has been proposed for the autoassembling of peptides scarcely soluble in aqueous solutions. Usually dimethylsulfoxide is considered as the ideal organic solvent due to its compatibility with water and low cytotoxicity. In this way, assembly and cell encapsulation can be produced when the peptide solution in the organic polar solvent is mixed with the cell culture medium [140,141].

Efforts have also been focused to develop light-sensitive hydrogels despite the fact that sol-gel transitions under UV irradiation may be harmful to cells. Therefore, nowadays systems mainly use UV before introducing the cells to the hydrogel. For instance, UV can be employed to produce channels in a formed hydrogel that could then be filled by the sol containing the cells [142]. Formation of a hydrogel can also be triggered by the action of enzymes able to form a gelator molecule from a given precursor. Usually this is characterized by the presence of a hydrophilic moiety that is susceptible to enzyme-catalyzed cleavage [143].

**Figure 7:**
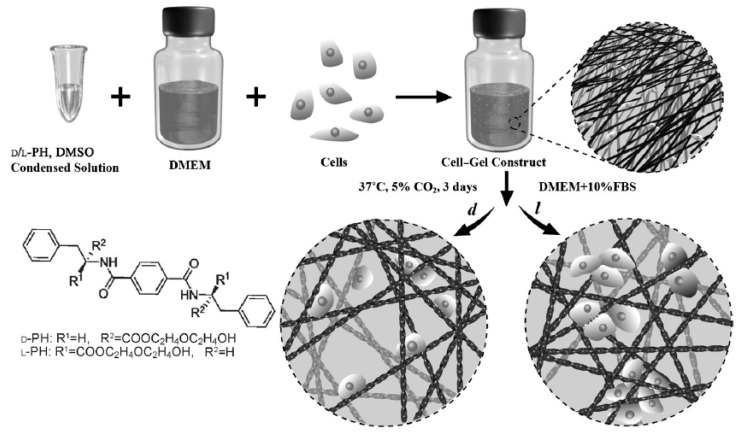
Schematic representation of the culture of fibroblast or endothelial cells in enantiomeric nanofibrous hydrogels (*d*, *l* right-handed and left-handed helices, respectively). Reproduced with permission from [144], copyright 2014 Wiley.

The use of peptides with chiral centers allows for obtaining chiral nanofibrous hydrogels that are influenced by adhesion and cell proliferation events. Specifically, two enantiomers of a 1,4-benzenedicarboxamide phenylalanine derivative have been employed as supramolecular gelators that produced left- and right-handed helices (Figure 7) [144]. The cell densities of fibroblast and endothelial cells in left-handed hydrogels were found to be twice those determined for hydrogels based on right-handed helices. Stereospecific interactions between chiral nanofibers and fibronectin were responsible for the observed effect. 

## 7. Micro- and Nanogels

Gelling biopolymers as proteins and polysaccharides can be intramolecularly crosslinked to produce small particles [145] with a globular structure when the concentration of the polymer is moderate and specifically lower than that employed to produce macroscopic gels [60]. These particles can be classified into microgels and nanogels when their diameter is lower than 0.5 μm or between 0.5–5 μm, respectively. These hydrogel particles are receiving increasing attention for rheology control, drug encapsulation, and targeted delivery [146]. Furthermore, particles can swell and deflate in response to external stimuli (e.g., pH, temperature, ionic strength) and consequently are appropriate for use in controllable and responsive systems. Cellulose, chitosan, gluten, soy protein, corn zein, casein, whey protein, gelatin, and collagen are the natural polymers most often employed for the preparation of these micro/nanogels [147]. 

**Figure 8:**
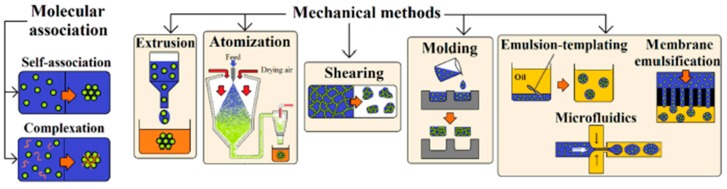
Scheme showing the different preparation methods applied for the production of micro/nanogel particles. Reproduced with permission from [147], copyright 2017 Elsevier.

Different techniques have been applied to get small gel particles, as indicated in Figure 8. These involve a typical phase separation caused by coacervation and desolvation, as well as processes induced by mechanical methods. 

A simple coacervation process implies only one polymer, whose molecular association may be favored, for example, by temperature or pH changes or by the use of a salt with higher affinity to the solvent (e.g., water) than the polymer itself. A typical example is the preparation of chitosan microgels by the addition of sodium hydroxide to a polymer solution also having a crosslinking agent (e.g., glutaraldehyde) [148]. Self-association of proteins can also be obtained by typical thermal denaturation processes as well as by cooling preheated protein solutions. In this case, unfolding took place in the heating step, giving rise to protein filaments that are able to be associated during the cooling step under appropriate pH and ionic strength conditions. Microgels based on soy protein (28–179 nm) were, for example, prepared by the addition of calcium cations, which favored the establishment of bridges between protein chains [149].

Protein microgels prepared by thermal denaturation are mainly based on the establishment of hydrophobic interactions. Basically, spherical particles (with diameters close to 50 nm) and strands (diameter less than 10 nm and length up to tens of microns) can be formed during denaturation, with the second morphology being favored when strong electrostatic repulsions exist. In a second aggregation step, fine strands or particulate gels can be derived, as has been reviewed by Nicolai and Durant [150]. pH and ionic strength were found to have a high influence on final morphology [151], as well as the protein concentration, temperature, and heating time.

Two polymers with opposite charges are required for the named complex coacervation process or associative phase separation. This process appears ideal for the preparation of gel particles composed of proteins and negatively charged polysaccharides. For example, ovalbumin is a protein that can be gelled by heat treatment and gives rise to nanogel particles where CS could subsequently be entrapped through electrostatic interactions [152]. 

A mechanical device can also be employed to form particles that will then be gelled through a physicochemical process. These mechanical methods were well explained by Farjami et al. [147] and include extrusion, atomization, shearing, emulsion, and micromolding processes.

The extrusion method is based on the flow of a polymer solution through a syringe needle, with the formed droplets hardened in a solution having crosslinking agents (e.g., enzymes and glutaraldehyde), by a temperature change (as in the case or gelatin), or by forming a complex with other polymers [153].

An aqueous solution of the selected biopolymers can also be atomized into a stream of hot air that evaporates the solvent. The resultant spray-dried particles can subsequently be rehydrated to form the corresponding gel particles. Particle sizes can be modified by using different gelling polymers and agents as well as controlling the speed of the process (gel particles or a continuous gel can be obtained under fast and slow gelation conditions, respectively). Collagen and carrageenan are good examples of microgels prepared under this atomization technology [154].

Application of a shear to a biopolymer gel system may induce its breakage and form irregular gel particles. The process has some advantages related to the facility of modulating the final properties (i.e., by controlling the shear and thermal history) [155]. 

Water-in-oil emulsions can be employed as templates where polymers dissolved in the oil phase are subsequently cross-linked. This technique has been successfully applied for the preparation of microgels able to encapsulate microorganisms and drugs, as is the case of those based on casein [156] and whey proteins [157], chitosan [158], and protein/sugar conjugates [159]. In the case of chitosan, the cross-linking process can also be completed easily by the addition of anionic agents such as sulfates or citrates, but in general the derived morphologies were irregular. The incorporation of gelatin allowed an improvement in the ionic crosslinking process and gave rise to regular, spherical and smooth microspheres with diameters in the micrometer range [160].

Preparation of anisotropic particles with complex architectures is also interesting for innovative applications like Janus motors [161]. The use of capillary flow-based approaches (e.g., microfluidics) has been revealed to be highly effective for producing polysaccharide hydrogel hetero Janus microparticles [162]. By contrast, drug delivery applications require the use of uniform particles in order to guarantee good repeatable release behavior. The use of a membrane is a methodology that allows for getting uniformly sized particles since the polymer aqueous solution should permeate through a membrane with a well-controlled pore size before being emulsified in the oil phase. This technique has been applied to produce uniform chitosan microspheres for drug delivery, taking advantage of its excellent mucoadhesive character and good permeability through biological surfaces. Thus, particles with a highly uniform diameter close to 0.4 mm have been obtained and applied to encapsulate proteins such as bovine serum albumin [163] and peptides like insulin [164]. 

**Figure 9:**
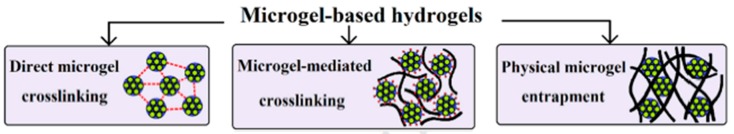
Scheme showing the different preparation methods applied for the production microgel networks. Reproduced with permission from [147], copyright 2017 Elsevier.

Figure 9 shows three methods that are usually applied to form networks from the nano/microgel particles: (a) direct cross-linking via physical or chemical interactions [165]; (b) reaction of functionalized microgel particles (e.g., having peroxy groups) with reactive polymers or even by grafting polymerization of monomers [166]; and (c) physical entrapment of hydrogel microparticles [167]. Microgels can, for example, be immobilized within the network produced by an in situ gelling hydrogel. Therefore, administration of the hydrogel is facilitated, while sustained or prolonged drug release can be achieved when the targeted drug is loaded in the entrapped particles. 

## 8. Shape Memory Hydrogels

Shape memory polymers (SMPs) are extensively studied due to their capability to fix one or more temporary shapes and recover their permanent shape under the effect of an external stimuli [168,169,170]. These properties (i.e., dual and multi shape memory effects) pave the way for the development of attractive new materials for applications in the biomedical field, aerospace, textiles, etc. [171,172]. Thermo-responsive SMPSs are probably the most usual systems and are specifically based on crosslinked polymers that adopt a temporary shape by vitrification or crystallization, recovering their permanent shape after heating [168,169]. 

**Figure 10:**
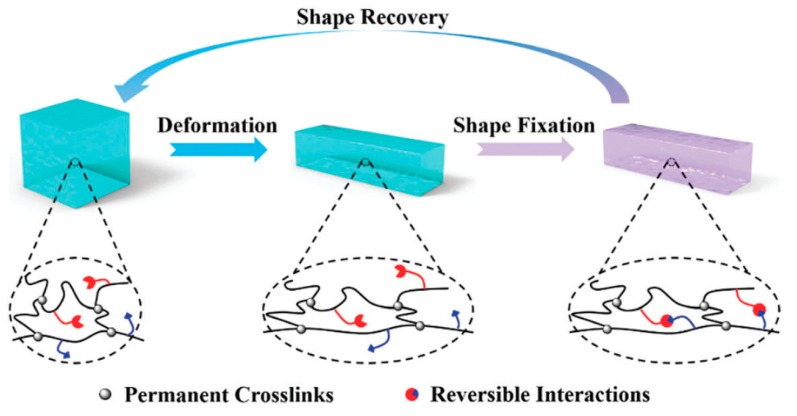
Mechanism of SSMHs: A crosslinked hydrogel can be deformed under an external stress and the temporary shape fixed by an external stimulus that induces the establishment of reversible interactions. A second stimulus may break the interactions and the material reverts to its permanent shape. Reproduced with permission from [173], copyright 2017 RSC.

Efforts are nowadays also focused on developing shape memory materials based on the establishment of supramolecular interactions and dynamic hydrogen bonds [173] (Figure 10). These supramolecular shape memory hydrogels (SSMHs) have the advantage of providing materials responsive to a wide range of external stimuli at body or ambient temperature since reversible interactions are usually multiresponsive [174].

A β-cyclodextrine modified CS has been employed as a SSMHs material taking advantage of the capability of the hydrophobic internal cavity of cyclodextrine to accommodate guest molecules such as ferrocene. Specifically, this supramolecular hydrogel was formed by the crosslinked CS derivative and a ferrocene-modified hyperbranched poly(ethylene imine) [175]. The derived host–guest interactions were redox-sensitive, being the temporary and permanent shapes achieved in the reduced and oxidized states, respectively. 

Photoactive gels have also been developed since light is a non-destructive and easily controllable stimuli. Therefore, photochromic groups (e.g., azobenzene) have been incorporated into functional gels by means of the supramolecular approach [176,177]. Host–guest inclusion complexes can be formed between *trans*-azobenzene groups and cyclodextrines, whereas steric repulsions are dominant when a bulky *cis* conformation is preferred. Therefore, systems based on an amphiphilic dendron with three L-glutamic acid units and an azobenzene moiety have been considered to form hydrogels susceptible to photo-triggered changes [178]. 

## 9. Molecularly Imprinted Hydrogels

**Figure 11:**
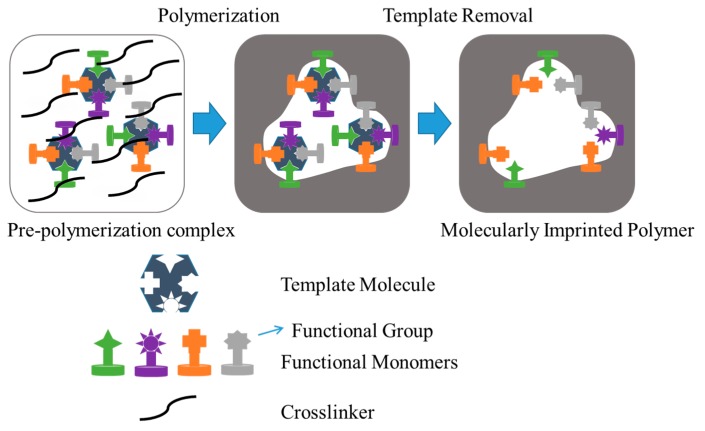
Scheme showing the different steps involved in the molecular imprinting process: Mixing of the appropriate template molecule and the selected functional monomer(s) and cross-linker(s) in a solvent; the polymerization of the formed complex; and finally the removal of the template, unreacted monomer, and cross-linker molecules. Adapted from [179].

Bioactive scaffolds for tissue engineering applications can be prepared by molecular imprinting, as recently reviewed by Neves et al. (2017) [179]. This technology has the great advantage of providing molecular memory effects when appropriate intelligent materials are selected [180,181]. Basically, a template molecule is combined with a functional monomer and finally cross-linked to create a polymer network. After removal of the template, the final material has ideal functionalization, cavity size, and shape to act specifically towards specific molecules (Figure 11). This high bioactivity and recognition ability make molecular imprinting a highly promising tool for tissue engineering.

Hydrogels have great potential for macromolecular imprinting since they facilitate both the movement of high molecular weight templates and the production of reversible systems sensitive to external stimuli (e.g., pH or temperature). In addition, they can be easily processed in different forms (e.g., sheets, coatings, or capsules). The main problems with such imprinting hydrogels concern the distortion of binding sites as a consequence of their easy expansion and contraction. Efficiency is consequently affected but different works have been focused on the development of tissue engineering applications that extend their most common use as drug delivery systems. 

BSA [182,183] and fibronectin (Fn) [184,185], a high molecular weight protein with wound healing and tissue repair activities, have been successfully employed as template molecules for alginate-based hydrogels. 

CS has also been considered to create pH-, temperature- and ionic-strength-sensitive hydrogels able to recognize, for example, the dipeptide of carnosine [186], an antioxidant that is found in muscle and brain tissue. Most commonly, chitosan has been combined with acrylamide monomers to form hydrogels that can also be used in biosensing applications such as the recognition of hemoglobine [187]. Systems based on a mixture of alginates and CS have also received attention due to the combination of oppositely charged functional groups (e.g., carboxyl and amine for alginate and CS, respectively), which support the interaction of the hydrogel with differently charged domains of proteins. The imprinting effect of the alginate/CS combination has effectively been demonstrated for BSA, lysozime, hemoglobin, and ovoalbumine proteins [188]. 

Cell adhesion on a particular substrate can also be improved by imprinting morphological and topographic features of cells (i.e., cells are considered the templates, as an extension of the abovementioned macromolecules). Despite its high potential, the technique is still in development and therefore mainly the more elemental acrylamide hydrogels have been assayed. Promising results have been attained with both epithelial and fibroblast cell lines [189]. 

## 10. Protein-Based Hydrogels for 3D Printing

**Figure 12:**
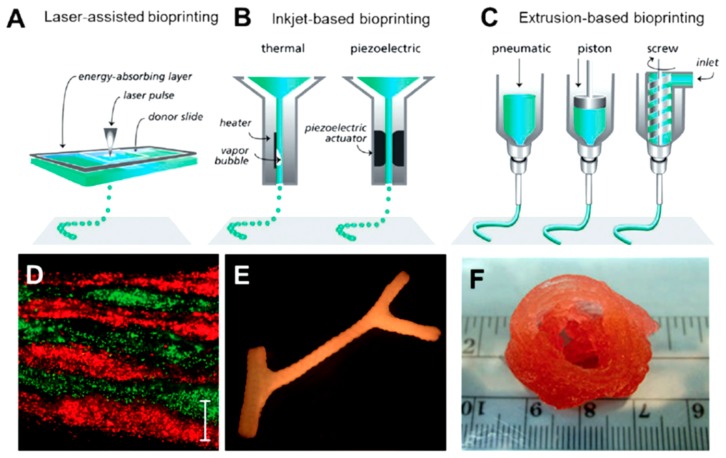
Main 3D bioprinting strategies: (**A**) laser-assisted; (**B**) injet-based and (**C**) extrusion-based. Examples of bioprinting tissues correspond to skin prepared by laser printing (**D**); branched vasculature obtained by inkjet printing (**E**) and heart aortic valve by extrusion bioprinting (**F**). Reproduced with permission from [194], copyright 2016 Wiley.

Three-dimensional printing technologies are a focus of increasing interest in the preparation of scaffolds based on the most important structural proteins (e.g., collagen, fibrin, silk, and even their composites with hydroxyapatite). Excellent reviews detail the printing parameters and physical properties of bioinks as well as the biological applications of printed scaffolds [190,191,192,193,194,195]. Three-dimensional printing technology allows for preparing complex scaffolds where the distribution of the different components plays a key role in their final performance. Furthermore, scaffolds can be designed in complex shapes at a reasonable cost, avoiding the use of expensive molds. Artificial tissues can easily be engineered through 3D bioprinting by precise control over spatial and temporal distribution of cells and the extracellular matrix components. Cells can be incorporated into the scaffolds following two approaches: printing a hydrogel precursor containing the selected cells [196] or depositing cells in the printed gel in a second step [197]. Processing conditions must be carefully selected in order to avoid cell damage when direct printing of embedded cells is performed [198]. Attention is nowadays given to three main bioprinting methodologies/strategies (Figure 12): inkjet-based, laser-assisted, and extrusion-based bioprinting [199]. The first methodology is based on the deposition of ink drops on successive layers, it being possible to place cells and proteins onto targeted spatial positions. This layer-by-layer technique is characterized by high resolution, reproducibility, and inexpensiveness [200]. In the second methodology, a pulsed laser source is able to transfer heat and eject a cell suspension from a coated absorption layer toward a substrate. Small volumes (from 10 to 7000 pL) of cell suspension can be printed with high resolution [201]. Cells or proteins are encapsulated in a hydrogel when the extrusion-based bioprinting is applied. In this case, a syringe, micronozzle, and pressure system are required to apply the hydrogel onto the substrate according to a specific 3D design. The process allows obtaining constructions with a relevant shape and size [202]. 

Water-soluble polymers able to form hydrogels are ideal components for the bioinks used in bioprinting technologies. The main advantages correspond to the possibility of controlling the gelation process and a favorable environment for cell growth [193], with poor mechanical properties and low dimensional stability as the main problems [199]. In any case, materials should contribute to: (a) an acceptable printing time, which is mainly influenced by the cross-linking kinetics and the rheological properties; and (b) a balanced cross-linking density that guarantees mechanical stability as well as the movement and proliferation of cells [203]. 

Collagen has been processed by the three aforementioned technologies, concerning in general the main problem of the mechanical stability of the constructions. Therefore, the use of salts and fillers has been proposed as well as the development of core/shell structures. In this case, alginate has been employed as the shell component since it can provide mechanical support to the collagen core after being cross-linked by the application of a CaCl_2_ aerosol solution [204]. Treatment of printed scaffolds with bicarbonate solutions increased the gelation rate while retaining the original shape and avoiding shrinkage or swelling effects [205]. 

Fibrin is another well-studied structural protein for preparing bioprinted scaffolds. Fibrin matrices display good mechanical properties and fast gelation and promote tissue regeneration and cell proliferation [206,207]. A layer-by-layer printing process was, for example, applied to produce scaffolds with embedded neurons. Specifically, layers of gelled fibrin were prepared from fibrinogen and thrombin and then neurons were printed on top of the layer. The 3D neural scaffold was attained after repeating the alternate bilayer printing five times. The final scaffold showed a modulus as high as 2.92 MPa, tensile strength of 1.7 MPa, and neurite overgrowth after 12 days of culturing [208].

Collagen/fibrin mixtures are also considered since they combine the high biocompatibility of collagen with the fast gelation rate of fibrin. The derived printed scaffolds showed faster wound closure and vascularization than hydrogels based only on collagen [209].

## 11. Conclusions

Cellulose and chitin are the two most abundant natural polymers on earth and consequently the use of them or their derivatives (e.g., chitosan) is widely studied. Biodegradability, biocompatibility, and renewability are characteristics that enhance their interest for biomedical applications and specifically for the development of hydrogels useful for tissue engineering, wound dressing, and drug delivery systems. Advantages related to processability, the presence of functional groups, and bioactivity have also shifted the research focus to improving solubility and basic properties and also to developing new physical and chemical cross-linkers. Hydrogels based on proteins are of great interest for use in physiological environments, taking into account the maintenance of their bioactive properties (e.g., the capability of collagen to enhance cell proliferation and tissue reconstruction). Hybrid materials based on their mixtures and the combination with other natural proteins such as collagen and gelatin may provide materials with considerable advantages over the individual components. Opportunities to create natural hydrogels are clear, although effort is still necessary to improve the final performance and get effective nontoxic cross-linkers. In this sense, the development of injectable hydrogels able to form in the body without requiring surgery or the development of stimuli-responsive smart materials is a clear example of the present trends. Double network hydrogels are highly promising to improve mechanical performance since they intrinsically have toughness and high resistance to mechanical stress due to the capacity for establishing entanglements that involve different network structures with efficient energy dissipation. The mechanical performance of hydrogels can be improved by the incorporation of fillers, with cellulose nanocrystals and hydroxyapatite being some of the natural compounds most often employed. 

The use of hydrogelators based on peptides has great potential in the biomedical field since they can mimic extracellular matrices. The advantages of such systems concern biocompatibility, controllable self-assembly, and the feasibility of creating hydrogels under physiological conditions through the establishment of physical interactions (e.g., hydrogen bonds, π–π stacking, etc.). In fact, hydrogen bonds are essential in living systems and may allow the culture of cells by means of proper stimulus (e.g., pH, light, enzymes, etc.). Furthermore, the properties of the derived hydrogels can be easily varied due to their dependence on the amino acid sequence, it being possible to get complex responses to external stimuli. Nevertheless, research must continue to address different challenges such as limited mechanical properties or the control over the porosity and morphology of the derived hydrogel. 

Micro- and nanogels have great potential as drug delivery systems and can be used in other areas as bioimaging, sensors, and even tissue engineering. The main applications are based on the ability to tune the particle size from the micrometric to the nanometric scale, together with the large surface area, which makes bioconjugation feasible, and the inner network that favors efficient encapsulation of molecules. In this sense, hydrogel particles show clear advantages over typical systems based on micelles and liposomes. 

Shape memory hydrogels can be designed to switch their form in response to external stimuli as heat, pH, light, or ions. Temporary cross-links can be modified and dual or even more complex effects can be derived. Recent advances are focused on achieving the appropriate functionality to satisfy the increasing demand for materials for the biomedical field and even for their use as smart actuators. 

Hydrogels appear to be ideal systems for the molecular imprinting of biomacromolecules and the production of bioactive matrices with recognition ability. Promising results as cell culture systems have specifically been attained using hydrogels based on natural polymers. 

Three-dimensional printing technologies are a major area of development, given that they are probably the most promising devices focused on biomaterial deposition. In addition, applications in tissue engineering and medicine are well documented, and the possibility of producing artificial organs justifies the great research efforts. Different examples emphasize the potential of the technology for bioprinting natural proteins despite the difficulty of preserving their structure and functionality. Recent efforts have demonstrated the feasibility of employing ink that incorporates cells as well as the possibility of culturing cells within the printed scaffolds.
